# Development, Validation, and Comparison of Two Mass Spectrometry Methods (LC-MS/HRMS and LC-MS/MS) for the Quantification of Rituximab in Human Plasma

**DOI:** 10.3390/molecules26051383

**Published:** 2021-03-04

**Authors:** Aurélien Millet, Nihel Khoudour, Dorothée Lebert, Christelle Machon, Benjamin Terrier, Benoit Blanchet, Jérôme Guitton

**Affiliations:** 1 Biochemistry and Pharmacology-Toxicology Laboratory, Lyon-Sud Hospital, Hospices Civils de Lyon, F-69495 Pierre Bénite, France; aurelien.millet@chu-lyon.fr (A.M.); christelle.machon@univ-lyon1.fr (C.M.); 2 Inserm U1052, CNRS UMR5286 Cancer Research Center of Lyon, F-69000 Lyon, France; 3 Department of Pharmacokinetics and Pharmacochemistry, Cochin Hospital, AP-HP, CARPEM 75014 Paris, France; nihel.khoudour@aphp.fr (N.K.); benoit.blanchet@aphp.fr (B.B.); 4 Promise Proteomics, 7 Parvis Louis Néel, F-38040 Grenoble, France; dorothee.lebert@promise-proteomics.com; 5 Analytical Chemistry Laboratory, Faculty of Pharmacy ISPBL, University Lyon 1, F-69373 Lyon, France; 6 Department of Internal Medicine, National Referral Center for Rare Systemic Autoimmune Diseases, Assistance Publique Hôpitaux de Paris-Centre (APHP-CUP), University of Paris, F-75014 Paris, France; benjamin.terrier@aphp.fr; 7 INSERM U970, PARCC, Université de Paris, F-75006 Paris, France; 8 UMR8038 CNRS, U1268 INSERM, Faculty of Pharmacy, University of Paris, PRES Sorbonne Paris Cité, CARPEM 75006 Paris, France; 9 Toxicology Laboratory, Faculty of Pharmacy ISPBL, University of Lyon 1, F-69373 Lyon, France

**Keywords:** rituximab, quadripolar mass spectrometer, albumin depletion, pharmacokinetics, orbitrap mass spectrometer, IgG-immunocapture

## Abstract

Rituximab is a chimeric immunoglobulin G1-kappa (IgG1κ) antibody targeting the CD20 antigen on B-lymphocytes. Its applications are various, such as for the treatment of chronic lymphoid leukemia or non-Hodgkin’s lymphoma in oncology, and it can also be used in the treatment of certain autoimmune diseases. Several studies support the interest in therapeutic drug monitoring to optimize dosing regimens of rituximab. Thus, two different laboratories have developed accurate and reproductive methods to quantify rituximab in human plasma: one using liquid chromatography quadripolar tandem mass spectrometer (LC-MS/MS) and the other, liquid chromatography orbitrap tandem mass spectrometer (LC-MS/HRMS). For both assays, quantification was based on albumin depletion or IgG-immunocapture, surrogate peptide analysis, and full-length stable isotope-labeled rituximab. With LC-MS/MS, the concentration range was from 5 to 500 µg/mL, the within- and between-run precisions were <8.5%, and the limit of quantitation was 5 µg/mL. With LC-MS/HRMS, the concentration range was from 10 to 200 µg/mL, the within- and between-run accuracy were <11.5%, and the limit of quantitation was 2 µg/mL. Rituximab plasma concentrations from 63 patients treated for vasculitis were compared. Bland–Altman analysis and Passing–Bablok regression showed the interchangeability between these two methods. Overall, these methods were robust and reliable and could be applied to routine clinical samples.

## 1. Introduction

CD20 is a glycosylated transmembrane phosphoprotein expressed on the surface of pre-B and mature B-lymphocytes, as well as many B-cell malignancies. Rituximab (RTX) is a chimeric IgG1κ therapeutic monoclonal antibody (mAb) that targets the CD20 antigen on B-lymphocytes [1]. It marks B-cells for destruction through direct induction of B-cell apoptosis, antibody-dependent cell-mediated cytotoxicity, or complement-mediated cytotoxicity. In the oncology field, it is used for the treatment of chronic lymphoid leukemia and diffuse and follicular non-Hodgkin’s lymphoma [2]. By depleting normal B-cells, rituximab also reduces the adaptive immune response against self. It is approved in the treatment of autoimmune diseases such as rheumatoid arthritis, granulomatosis with polyangiitis, and moderate to severe pemphigus vulgaris. Finally, it has also shown benefit in treating some other autoimmune diseases such as vasculitis [3]. Different factors such as constant fragment gamma (Fcγ) and CR3 polymorphisms, gender, rituximab pharmacokinetics are known to contribute to highly variable clinical response in patients treated with rituximab [4]. The interindividual variability in rituximab systemic exposure is usually large because of differences in antigenic burden, which can influence rituximab clearance [5]. Besides, several studies reported a relationship between rituximab plasma concentrations and efficacy in patients treated for lymphoproliferative disorders [6]. These results support the use of therapeutic drug monitoring to optimize dosing regimens of rituximab in oncology. In contrast, pharmacokinetic/pharmacodynamic data are sparse in patients treated for autoimmune diseases [7,8], which deserves more investigation in the future.

For a few years, works on mAbs quantifying methods are in full swing. Regarding RTX, some ELISA methods [9,10,11], the Gyrolab™ assay [12], and mass spectrometry methods [13,14,15,16,17] have been described for their quantification in blood. The mass spectroscopy (MS) method developed by Mills et al. was based on the quantification of the light chain of RTX after disulfide bonds reduction and using quadrupole time of flight (Q-TOF) detection and vedolizumab as an internal standard (I.S.) [13]. Another method was developed with a sample preparation consisting of methanol precipitation, followed by reduction, peptide digestion, and solid-phase extraction [14]. Detection was achieved using a Triple TOF mass spectrometer, and the labeled peptide was used as an I.S. Recently a quadripolar tandem mass spectrometer method was published with a sample preparation by IgG-immunocapture and digestion and stable-isotope-labeled-adalimumab as an I.S. [15]. The analytical performances of the methods based on MS are quite similar: the ranges of the calibration curves were from 1 to 200 µg/mL [14,15], from 0.586 to 300 µg/mL [16], or from 5 to 100 µg/mL [17]. All the MS methods have an accuracy and precision < 15%. The Gyrolab™ assay was found to have a dynamic range from 0.09 to 60 µg/mL [12] and ELISA from 0.5 to 800 µg/mL[11], from 6.6 to 3400 µg/mL [9], or from 2 to 50 µg/mL for a commercial kit [18]. With immunological methods, the precision and accuracy were below 15% [12,18] or 25% [9,11].

This article describes the development and validation of two RTX quantification methods performed in two separate laboratories, one using a quadripolar tandem mass spectrometer and the other a liquid chromatography orbitrap tandem mass spectrometer (LC-MS/HRMS) (Orbitrap™). In both cases, a full-length stable isotope-labeled RTX was used as an I.S., and two samples preparation (albumin depletion and IgG-immunocapture) were performed. Several comparisons using plasma samples from 63 patients treated for vasculitis were carried out based on the surrogate peptide selected, mass analyzers, and sample preparations.

## 2. Results and Discussion

### 2.1. Selection of Proteotypic Peptides

**Figure 1:**
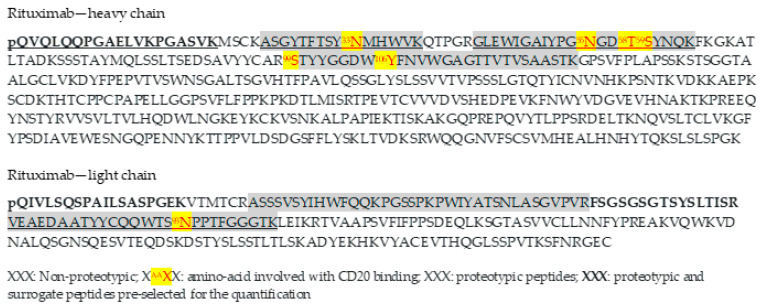
Amino-acid sequences of the heavy-chain (HC) and light-chain (LC) of rituximab (RTX).

Since RTX is a chimeric monoclonal antibody, several tryptic peptides are proteotypic and could be used as surrogate peptides for quantification. Thus, from the in-silico study, eight candidate peptides were found (Figure 1).

**Figure 2:**
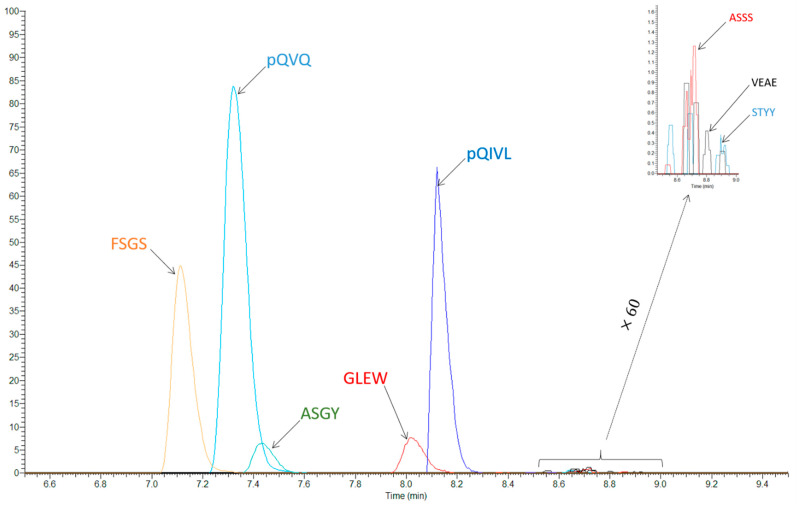
Liquid chromatography orbitrap tandem mass spectrometer (LC-MS/HRMS) chromatogram of proteotypic peptides obtained from a pure solution of RTX at 10 µg/mL analyzed in Full Scan Mode.

The selection of surrogate peptides was based on the abundance of the parent ions and the signal/noise ratio from spiked plasma, considering the matrix effect and the sample preparation recovery. Among the candidate, QVQLQQPGAELVK (QVQ or HC1) and QIVLSQSPAILSASPGEK (QIVL or LC1) peptides were pre-selected with both MS methods because the abundance of the parent ions was clearly higher (Figure 2).

**Figure 3:**
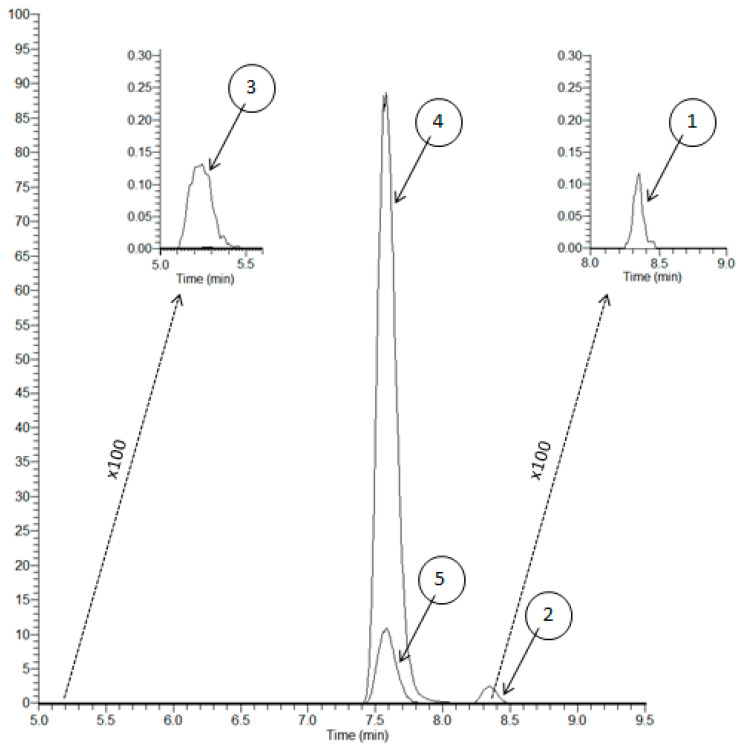
Chromatograms of the different forms of QVQLQQPGAELVK peptide after trypsin digestion of a pure solution of rituximab. ①: Wild type peptide: *m/z* = 719.4066 (charge: +2); ② N-terminal Pyroglutamate (loss of NH_3_): *m/z* = 710.8934 (charge: +2); ③: Misscleavage (addition of PGASVK): *m/z* = 659.7091 (charge: +3); ④: Misscleavage (addition of PGASVK) and N-terminal pyroglutamate (loss of NH_3_): *m/z =* 980.5467 (charge: +2); ⑤: Misscleavage (addition of PGASVK) and N-terminal pyroglutamate (loss of NH_3_): *m/z* = 654.0336 (charge: +3).

These two peptides exist in different forms and with two different charge states, as shown for QVQ. The two peptides present the transformation of N-terminal glutamine in pyroglutamate (loss of NH_3_), and pQVQ also has a misscleavage before a proline generating the pQVQLQQPGAELVKPGASVK (pQVQ or HC1) peptide, which was the most abundant form (Figure 3). Ions with *m/z* +2 were the most abundant for pQVQ and pQIVLSQSPAILSASPGEK (pQIVL or LC1). The FSGSGSGTSYSLTISR (FSGS or LC6) was a third peptide only pre-selected for LC-MS/HRMS. Finally, pQILV was only selected as a qualifier peptide because an important ion suppression effect was measured for this compound (see below), pQVQ was used for the quantification with both MS methods and with LC-MS/HRMS, FSGS was also tested for the quantification.

None of the three peptides selected have amino-acids involved in the rituximab-CD20 bond [19]. The primary structure of three commercialized biosimilar peptides (PF-05280586, GP-2013, and ABP-798) were checked as being the same as RTX [20,21,22]. Thus, the two RTX princeps and the three biosimilar drugs could be quantified with the selected proteotypic peptides.

Previous studies also used pQVQ as a surrogate-peptide for quantification with quadripolar tandem mass spectrometer [15] or pQILV and GLEWIGAIYPGNGDTSYNQK, another proteotypic peptide, using a Triple TOF mass spectrometer [14].

### 2.2. Liquid Chromatography and Mass Spectrometry

**Figure 4:**
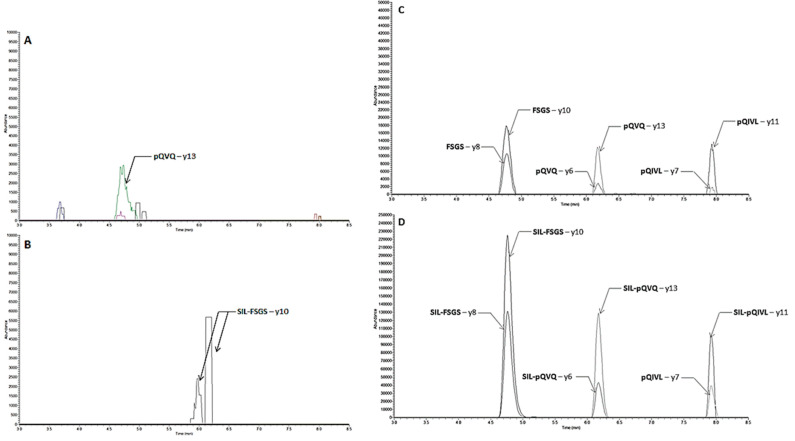
PRM chromatograms with LC-MS/HRMS assay of rituximab (**A**) and (**C**) and SIL-rituximab (**B**) and (**D**). **A** and **B**: blank sample; **C**: sample spiked at 2 µg/mL (lower limit of quantitation (LLOQ)); **C**: SIL-rituximab at 15 µg/mL.

**Figure 5:**
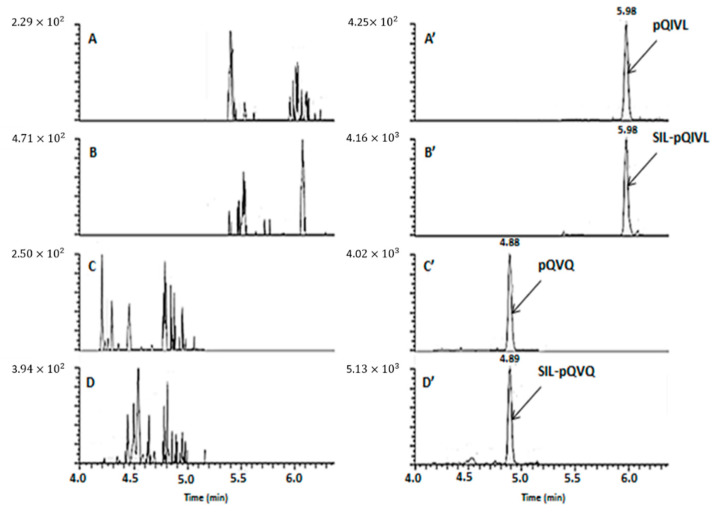
Multiple-reaction monitoring (MRM) chromatograms with LC-MS/MS assay of rituximab assay of rituximab (**A**), (**A’**) and (**C**), (**C’**) and SIL-rituximab (**B**), (**B’**) and (**D**), (**D’**). **A**, **B**, **C**, **D**: blank sample; **A’** and **C’**: sample spiked at 5 µg/mL (LLOQ); **B’** and **D’**: SIL-rituximab at 15 µg/mL.

Separation of the surrogate peptides was accomplished in 9.5 and 16 min with LC-MS/MS and LC-MS/HRMS, respectively. A step gradient was used with water and acetonitrile, each containing 0.1% formic acid. Representative parallel reaction monitoring (PRM) chromatograms obtained with LC-MS/HRMS are shown in Figure 4. The retention times of the peptides FSGS, pQVQ, and pQIVL were 4.8 min, 6.2 min, and 7.9 min, respectively. Figure 5 shown MRM chromatograms obtained with LC-MS/MS, and the retention times of the peptides pQVQ and pQIVL were 4.9 min and 6.0 min, respectively. All compounds were resolved completely with a good peak shape.

Protonated molecules were predominantly formed, and the [M + H]^2+^ ion of each peptide was selected as the precursor ion to find the most abundant product ion. For the quantification with LC-MS/HRMS, y8 + y10 and y6 + y13 were used as the product ion for FSGS and pQVQ, respectively (Table 1). For LC-MS/MS, the sum of the product ion y6 + y10 + y12 + y13 from pQVQ was used for quantification (Table 2).

### 2.3. Result of Validation

#### 2.3.1. Selectivity

With LC-MS/HRMS, no interference on chromatograms of RTX or isotope-labeled rituximab (SIL-RTX) was observed from ten blank plasmas (Figure 4). With LC-MS/MS, the signal observed due to endogenous components in comparison to the signal at the lower limit of quantitation (LLOQ) (5 µg/mL) was from 0.3% to 8.3% and 0.8 to 4.8% for RTX and SIL-RTX, respectively (Figure 5). These results show an excellent selectivity with both mass spectrometry methods.

#### 2.3.2. Calibration, Accuracy, Precision, LLOQ, and Dilution

Quantification was based on the ratio of RTX and the SIL-RTX as an internal standard. Calibration curves were generated using a weighted (1/X) quadratic regression. With the LC-MS/MS, the LLOQ was set at 5 µg/mL (4.6 +/− 0.4 µg/mL), and the within-day, the between-day, and the accuracy were equal to 3.3%, 8.5%, and 91.1%, respectively. With the LC-MS/HRMS, the LLOQ was set at 2 µg/mL (2.1 +/− 0.2 µg/mL), and the within-day, the between-day, and the accuracy were equal to 7.5, 8.6, and 107.1%, respectively. The back-calculated calibration concentrations were within 91.8–103.1% (FSGS peptide) and within 88.4–102.1% (pQVQ peptide) of the theoretical value for LC-MS/HRMS (Table 3). With LC-MS/MS using (pQVQ peptide), the accuracy was within 94.1–108.2% (Table 4). The precision for standards was <12.6% (FSGS peptide) and <16.6 (pQVQ peptide) for LC-MS/HRMS and <11.3% for (pQVQ peptide) with LC-MS/MS.

In Table 5 and Table 6, a summary of the intra- and inter-day accuracy and precision assay performance is shown for quality control (QC) samples for both methods. All these parameters were <11.5% for LC-MS/HRMS and <10% for LC-MS/MS.

For LC-MS/HRMS, dilution integrity of RTX presented acceptable accuracy and precision after diluting1/5 (*v/v*) in blank plasma with a mean concentration equal to 524 +/− 46 µg/mL (bias 4.8% and reproducibility 9.1%).

#### 2.3.3. Matrix Effects, Carryover, and Sample Stability

Matrix effect due to endogenous compounds in a biological matrix is commonly encountered during the analysis by mass spectrometry in electrospray ionization (ESI) mode. The matrix effect varies greatly from one peptide to another among and according to the mass technology (Table 7). Since pQIVL exhibited the most important matrix effect with both MS methods, it was not selected for RTX quantification but only used as a qualifier peptide.

No peaks in the blank plasma samples were observed after three injections of the highest standard, indicating no carryover with both MS assays.

Pre-analytical stability tests did not show any degradation, either at −20 °C for quality controls (QCs) (Table 8) or after three freeze-thaw cycles from patient samples (Table 9). Furthermore, the post-analytical stability of RTX on autosampler (+4 °C) was checked (Table 8).

### 2.4. Application

As described, two MS methods for RTX quantification were validated in two different laboratories. In each laboratory, two sample preparations were performed (albumin depletion and IgG-immunocapture), and in all cases, a full-length stable isotope-labeled rituximab was used. From sixty-three plasmas from patients with vasculitis, several comparisons were conducted to evaluate the interchangeability between both MS assays.

**Figure 6:**
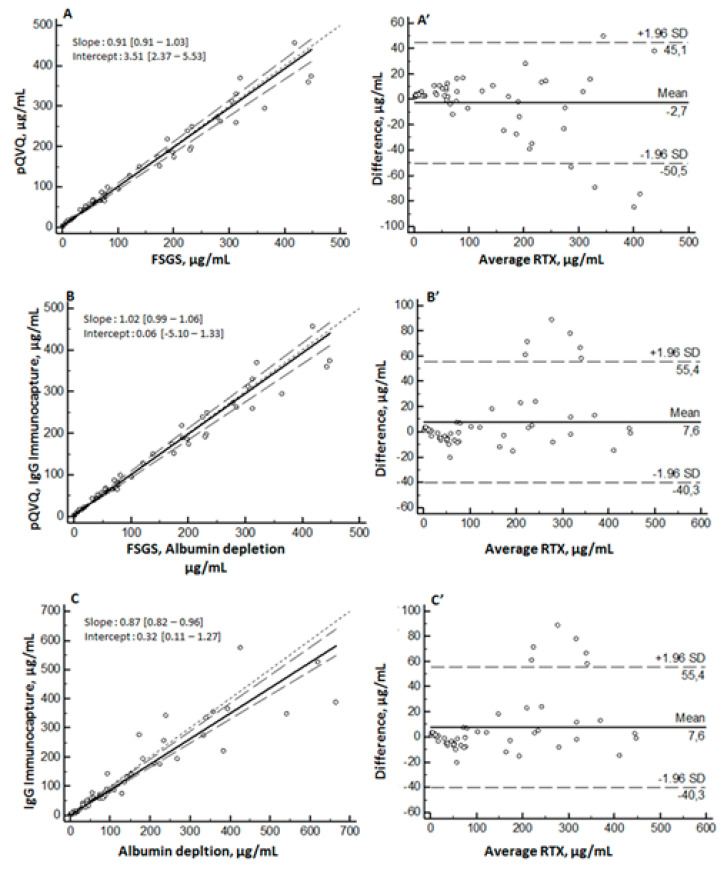
Intra-laboratory comparison of rituximab (RTX) quantification. Passing–Bablok regression (left), with solid line representing regression line and dashed line representing 95% confidence interval for regression line, and Bland–Altman difference-plot (right). Comparison between pQVQ and FSGS by albumin depletion protocol on the same extract sample (**A**), (**A’**); comparison between FSGS by albumin depletion and pQVQ with IgG-immunocapture (**B**), (**B’**) with LC-MS/HRMS and comparison between albumin depletion and immunocapture on pQVQ with LC-MS/MS (**C**), (**C’**). SD: standard deviation.

Three intra-laboratory comparisons are shown in Figure 6 (Passing–Bablok and Bland–Altman). On LC-MS/HRMS system, we compared results obtained from the two surrogate peptides on the same extraction and injection (Figure 6A) and results obtained from two different peptides analyzed on two different days with a different sample preparation protocol (Figure 6B). For LC-MS/MS, we compared results obtained from albumin depletion and IgG-immunocapture (Figure 6C).

**Figure 7:**
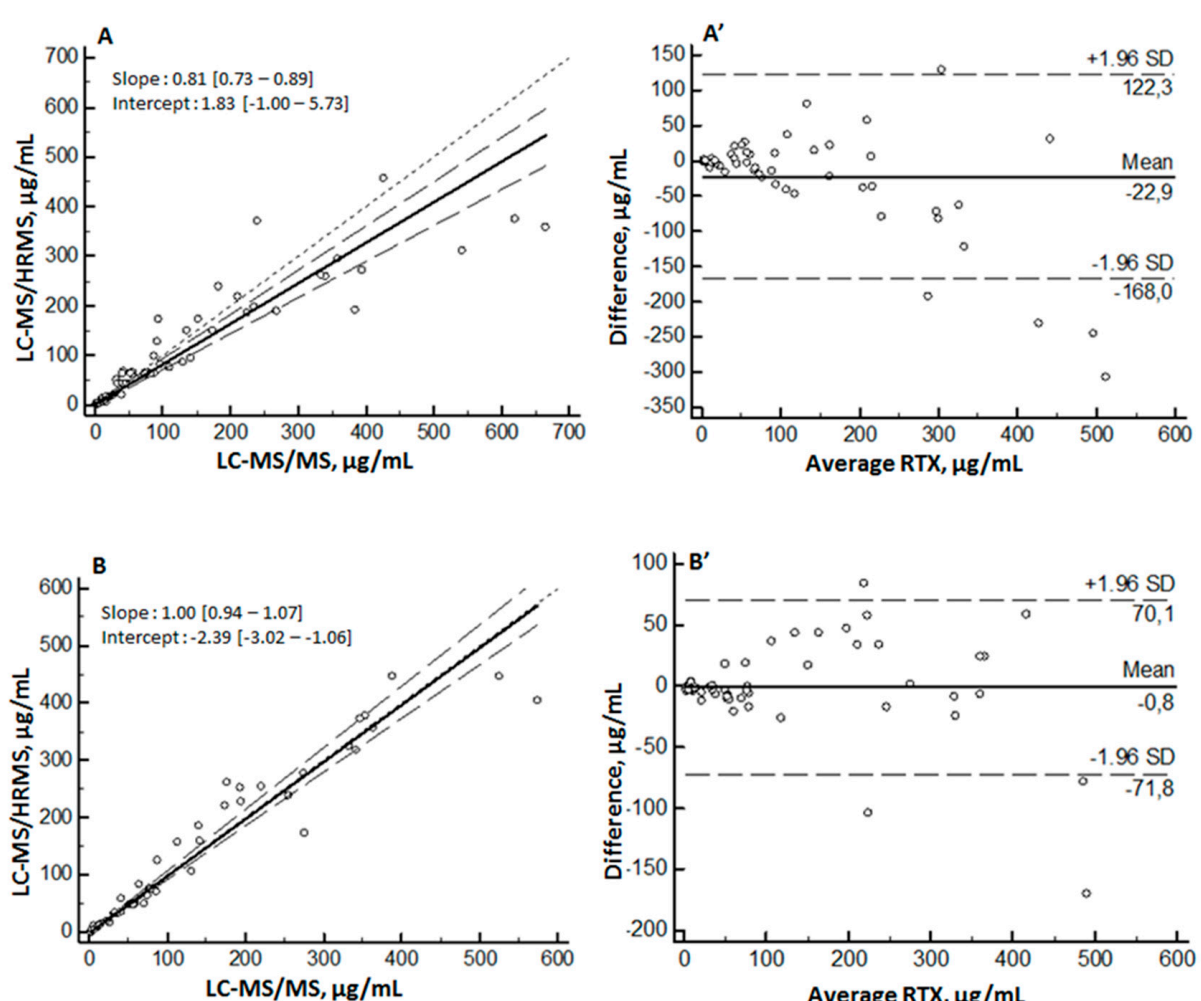
Inter-laboratory comparison of rituximab (RTX) quantification. Passing–Bablok regression (left), with solid line representing regression line and dashed line representing 95% confidence interval for regression line, and Bland–Altman difference-plot (right). Comparison between results obtained with the pQVQ and albumin depletion protocol on LC-MS/HRMS and LC-MS/MS (**A**), (**A’**) and comparison between results obtained with pQVQ and IgG-immunocapture protocol with LC-MS/HRMS and LC-MS/MS (**B**), (**B’**). SD: standard deviation.

We also compared inter-laboratory results, as shown in Figure 7. In all cases, the Bland–Altman analysis did not show any significant bias between the two methods. The Passing–Bablock linear regression analysis also showed a good agreement between the methods whatever the surrogate peptide used, the sample preparation, or the mass spectrometer. However, we note that the comparison between results obtained from albumin depletion (Figure 7A) was less correlated than the results obtained from IgG-immunocapture (Figure 7B).

This could be explained by the fact that IgG-immunocapture produces a less complex extract than albumin depletion. The preparation of samples by albumin depletion is faster (approximately 2 h) but required many manual steps. Conversely, carrying out the IgG-immunocapture is longer (approximately 4 h), but several phases consist of incubation, agitation, and evaporation times. Regarding the cost, albumin depletion is less expensive, but the choice of the sample preparation must also take into account the production time and the selectivity of the preparation.

### 2.5. Comparison with Previously Published MS Methods

The analytical performances of MS methods previously published for the quantification of RTX were quite similar to the performance of the two methods described here. The LOQ (expressed in ng injected on column) of the methods were 8 ng (LC-MS/HRMS) and 20 ng (LC-MS/MS), in the same range previous MS assays using LOQ, from 1.2 to 21.1 ng [13,14,15,16,17]. These values, according to the sample preparation, corresponded to a LOQ from 0.586 to 5 µg/mL. However, obtaining a very low LOQ was not a major issue for us because it was reported, from a study of 166 patients treated by RTX for lymphoma, a median serum level around 6 µg/mL and 25 µg/mL in non-responders and in responders was detected, respectively [6]. Excepted for an assay based on a middle-down analysis [13], the precision of published MS methods was below 15%, as was observed in the present work. Thus, the analytical validation parameters of mass spectrometry methods meet the required criteria of the validation guidelines. In the present study, two-sample preparation protocol were tested. Albumin depletion and IgG immunocapture were simple and were quite quick to perform since proteolysis did not need human manual interventions. A previous study describes a less time-consuming sample preparation method based on nano-surface and molecular-orientation limited (nSMOL) technology and the use of a high trypsin concentration; however, the cost for each RTX quantification will be more expensive and should be taken into account for routine activity [16]. Other studies proposed sample preparation requiring more steps with human interventions such as reduction-alkylation [13] or solid-phase extraction [14]. Here we present the first comparison of two RTX quantification methods based on two different mass detector technologies performed in two separate laboratories. Thus, all the process from the patient sample to the result was separately performed in each lab. Moreover, more than 60 sample patients were used to show the applicability of both methods, whereas some assays did not present application on patient samples [14,16,17]. Finally, the robustness of both MS methods and the relevance to use a full-length stable isotope-labeled RTX-like as an internal standard was demonstrated, with the main focus being on the inter-laboratory transferability, which is an important point for the development of therapeutic drug monitoring of RTX.

## 3. Materials and Methods

### 3.1. Chemicals and Reagents

The pure solution of RTX was Truxima^®^ (10 g/L, Celltrion Healthcare, Budapest, Hungary) for LC-MS/HRMS assay and Rixathon^®^ (10 g/L, Sandoz, Kundl, Austria) for LC-MS/MS assay. Full-length stable isotope-labeled rituximab (Arginine 13C6-15N4 and Lysine 13C6-15N2) (SIL-RTX) was purchased from Promise Advanced Proteomics (Grenoble, France). SIL-RTX presented with a purity > 95% and labeling > 99%. Stock solutions of RTX and SIL-RTX were prepared in water at 1 g/L and at 100 mg/L, respectively, and stored at +4 °C.

ULC/MS grade acetonitrile was obtained from Biosolve (Dieuze, France) and formic acid (FA) from Fisher Chemicals (Illkirch, France). Ultrapure water (resistivity 18.2 mΩ.cm) was obtained using a Milli-Q Plus^®^ system (Millipore, Molsheim, France). PBS buffer (pH 7.4, molarity 10X) was from Gibco (Thermo Fisher, Waltham, MA, USA). Trypsin Gold, Mass Spectrometry Grade were purchased from Promega (Madison, WI, USA). Propan-2-ol for analysis and trichloroacetic acid 20% for analysis were obtained from Carlo Erba Reagents (Val-de-Reuil, France). Ammonium bicarbonate for mass spectrometry was from Sigma-Aldrich (Saint-Quentin-Fallavier, France). Drug-free human plasma was provided by the regional blood service (EFS Rhône-Alpes and Ile-de-France, France). Low adsorption polypropylene microtubes from Dutsher (Brumath, France) were used throughout the study.

### 3.2. Chromatographic and Mass Spectrometric Conditions and Instrumentation

#### 3.2.1. LC-MS/HRMS

**Figure 8:**
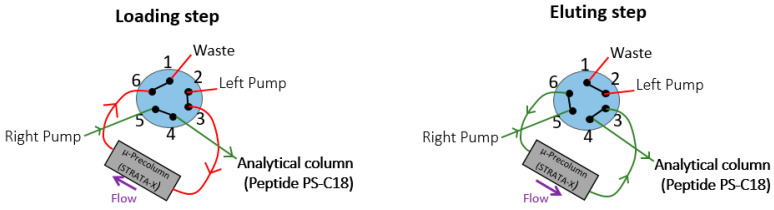
Switching valve used with the LC-MS/HRMS device to clean-up the sample.

The LC system was an UltiMate 3000 chromatographic system (ThermoScientific, USA) with two ternary pumps. An on-line SPE µ-Precolumn (Strata-X^TM^; 20 × 2.0 mm, 25 µm, Phenomenex, Torrance, CA) was used to clean up extracted samples before the chromatographic separation, which was performed on a bioZen^TM^ Peptide PS-C18 chromatographic column (100 × 2.1 mm, 1.6µm, Phenomenex, Torrance, CA). The mobile phase was composed of water (A) and acetonitrile (B), each containing 0.1% formic acid. Pump 1 delivered an isocratic mobile phase composed of 0.1% formic acid (FA) aqueous solution and acetonitrile (95/5; *v/v*) through Strata-X^TM^ at 150 µL/min. Pump 2 performed the following gradient: 0–1min, 15% B; 1–8 min, 15–52.5% B; 8–10 min, 90% B; 10–16 min, 15% B (A: water, B: acetonitrile, each containing 0.1% formic acid). The chromatographic column was heated at 50 °C. A six-port valve was used to switch from precolumn to analytical column and to inject in backflush mode the 20 µL sample (Figure 8). The sample compartment in the autosampler was maintained at +4 °C.

Detection was performed with a Q-Exactive Plus Orbitrap mass spectrometer (Thermo Scientific, Bremen, Germany) via a heated electrospray ionization source (HESI) interface in positive ionization mode with a spray voltage of 4.0 kV and a capillary temperature of 300 °C. High-purity nitrogen gas was employed as the sheath gas (30 arbitrary units (au)) and auxiliary gas (10 au). Quantification of RTX was performed by using Parallel Reaction Monitoring (PRM) mode at a resolving power of 35,000 at *m/z* 200. The precursor ions filtered by the quadrupole in a 1 *m/z* isolation window were fragmented in a higher-energy collisional dissociation (HCD) collision cell with a normalized collision energy (NCE) of 25 au and a nitrogen collision pressure of 1.5 mTorr. Product ions were detected in the Orbitrap mass analyzer at an AGC value of 1 × 106 and an IT of 128 ms.

#### 3.2.2. LC-MS/MS

LC device was a Vanquish system (ThermoScientific, USA) with a binary pump. The chromatographic conditions were close to those used for MS/HRMS method: the same column maintained at 50 °C and same mobile phase, but without the on-line extraction step. The gradient program, delivered at 400µL/min, was performed as follow: 0–1 min, 5% B; 1–6 min, 5–90% B; 6–7.5 min, 90% B; 7.5–9.5 min, 5% B (A: water, B: acetonitrile, each containing 0.1% formic acid). Extracted samples were maintained at +4 °C on the autosampler. The detector was a TSQ-Altis Triple Quadrupole mass spectrometer (Thermo Scientific, USA) with a HESI source. The instrument operated in positive ion mode, and the pressures for the nitrogen sheath gas, auxiliary gas, and sweep gas were maintained at 40, 10, and 1 au, respectively. Spray voltage and capillary temperature were set at 3.0 kV and 235 °C, respectively. The first (Q1) and the third (Q3) quadrupole were set with full-width at half maximum height of 0.7 Th. RTX was quantified in selected reaction monitoring (SRM) mode with a 20 ms dwell time. Argon was used as collision gas at 1.5 mTorr.

### 3.3. Selection of Peptides for Quantification

As described by El Amrani et al. [23], the first step to develop a quantification method of mAbs consisted of selecting signature peptides. For this purpose, an in silico digestion with Skyline^®^ software (https://skyline.ms/project/home/begin.view) (accessed on 23 December 2020) followed by the analysis of the uniqueness of generated peptides with BLAST^®^ software (http://blast.ncbi.nlm.nih.gov/Blast.cgi) (accessed on 23 December 2020) was completed. To define potential proteotypic peptides, we took into account in one hand that some peptides may undergo post-translational modifications, either on an amino-acid (methionine (di)oxidation, asparagine or glutamine deamidation, cysteine carbamylation) or such as a tryptic misscleavage, especially when an arginine/lysine was followed by a proline [24]. On the other hand, we also took into account that a transformation of N-terminal glutamic acid or glutamine in pyroglutamic acid (pE) or pyroglutamate (pQ) respectively may occur [25,26]. Then, to determine the most relevant peptides from an analytical point of view, samples obtain after trypsin digestion of pure solution of RTX and blank plasma were analyzed with LC-MS/HRMS and LC-MS/MS. The final selection of the peptides was based on the highest ratio signal/noise and the selectivity.

### 3.4. Sample Preparation

Validation of both methods and analysis of samples from patients were performed using a sample preparation based on albumin depletion adapted from the method described by Liu et al. [27]. To 10 µL of plasma (standard, quality control (QC) and patient samples), were added 20 µL of SIL-RTX at 15 µg/mL with LC-MS/HRMS and 25 µg/mL with LC-MS/MS in PBS and 300 µL of a mixture of isopropanol with 1% of trichloroacetic acid, in a low adsorption Eppendorf tube. After a brief vortexing step, eppendorfs were centrifuged at 2000· *g* for 5 min. After removing the supernatant, which contains albumin, a washing step with 200 µL of methanol was carried out to remove trichloroacetic acid residues, followed by a second quick centrifugation at 2000· *g* for 2 min. The supernatant was then removed, and the pellet was resuspended in 45 µL of ammonium bicarbonate (100 mM). Proteolysis was performed with 5 µL of Trypsin Gold at 0.2 µg/µL, and Eppendorfs were stored at 37 °C overnight. After centrifugation (13,000· *g*, 5 min), the clear supernatant was transferred in a vial to inject 20 µL into the liquid chromatography system.

Sample preparation based on IgG immunocapture (Pierce™ Protein G Spin plate) was also tested for sample patients. The plate was washed twice with 200 µL PBS (1×), and the buffer was discarded, 20 µL of sample was mixed with 80μL of PBS (1×) containing the SIL-RTX (30 µg/mL at final concentration). Incubation for 1 h was performed with an orbital shaker at room temperature. Then the resin was washed three times with 200 µL PBS (1×), and IgG elution was obtained by applying two times 150 µL of a mixture containing water/acetonitrile (50/50, *v/v*, and 0.1% FA). Centrifugation (1000· *g*, 1min) was performed, and fractions were combined, dried at room temperature, and the residue was resuspended with 45 µL of ammonium bicarbonate (100 mM). Trypsin Gold was added (1 µg/sample), and the mixture was incubated at 37 °C for 16 h. The digestion reaction was stopped by adding FA (final concentration at 1%), samples were centrifuged (5 min, 13,000· *g*), and 20 µL of supernatant were injected into the LC apparatus.

### 3.5. Method Validation

The selectivity of the methods was tested by analysis of six (LC-MS/MS) and ten (LC-MS/HRMS) blank plasma from patients not treated by RTX.

For the LC-MS/MS method, the calibration standard samples were prepared by spiking the blank plasma into concentrations 5–500 µg/mL (5, 10, 20, 50, 100, 250, and 500 µg/mL, three QCs were also prepared at 15, 75, and 300 µg/mL and SIL-RTX was added at a final concentration of 25 µg/mL. A total of 12 calibration curves (prepared as a single replicate and analyzed on 12 different days) were generated during the entire validation process. Ten runs included a calibration curve and QCs n three replicates.

For the LC-MS/HRMS method, the calibration standard samples were prepared by spiking the blank plasma into concentrations 10–200 µg/mL (10, 25, 50, 100, 150, and 200 µg/mL), three QCs were also prepared at 20, 80, and 160 µg/mL and SIL-RTX was added at a final concentration of 30 µg/mL. A total of 7 calibration curves (prepared as a single replicate and analyzed on 7 different days) were generated during the entire validation process. Five runs included a calibration curve and QC in six replicates.

In both cases, the accuracy and precision of the assays were assessed by the mean relative percentage deviation from the nominal concentrations and the within-run precision and between-run precision, respectively. The lower limit of quantitation (LLOQ) was tested at 2 µg/mL and 5 µg/mL for LC-MS/HRMS and LC-MS/MS, respectively. In both cases it was verified that the variance was within 20% for both precision and accuracy. For LC-MS/HRMS, the LLOQ was tested in triplicate on three different days, whereas for LC-MS/MS, the LLOQ was tested in duplicate on twelve different days. The upper limit of quantitation (ULOQ) was set as the concentration of the higher calibration standard. A dilution procedure was validated with LC-MS/HRMS method if the concentration from patient samples would be over the ULOQ. Thus, blank plasma was spiked with RTX at 500 mg/L and then diluted by 1/5 with plasma and analyzed in duplicate on three different days. The accuracy and precision should be <20%.

Carry-over was assessed by injection of three blank plasma samples after the highest calibration samples were also injected three times. This cycle was repeated twice. Peak area responses in the blank matrix samples were compared with the analyte area responses of the LLOQ of the method, and values ≤20% of the corresponding analyte response of the LLOQ level were considered acceptable.

The matrix effect was evaluated with both methods by analyzing extracted plasma spiked with RTX and compared to water samples spiked at the same final concentration. For LC-MS/HRMS, six different samples with 50 µg/mL of RTX were analyzed, and with LC-MS/MS four different plasmas at 15µg/mL (C1) and 300µg/mL (QC3) were processed. This experiment was performed on three different days.

Pre-analytical and autosampler stabilities were assessed with the LC-MS/MS method using the three levels of plasma QCs (low, medium, and high). The QCs were stored at –20 °C for 3 months and then re-analyzed. Stability on autosampler (+4 °C) was evaluated by re-analyzing the QCs samples 72 h after the first injection. The freeze-thaw stability was assessed with the LC-MS/HRMS method by re-analyzing seven samples of treated patients in triplicate following three cycles at −20 °C. In all cases, stability was confirmed if calculated bias was < +/−15% from the initial value and a precision of <15%.

### 3.6. Application and Method Comparison

Sixty-three patients with vasculitis were treated once monthly with RTX (Mabthera^®^ or Rixathon^®^). The RTX dose depended on the period treatment: 375 mg/m² during induction treatment and 500 mg/m² during maintenance treatment. Samples were collected during the Mainritsan trial and in the context of routine clinical care. Blood samples were collected at a steady-state into tubes containing lithium heparin just prior to the next infusion (trough concentration) or at the end of infusion (peak concentration). The samples were centrifuged for 10 min at 3000× *g*; then plasma was frozen (−20 °C) until assay.

Medcalc software (version 7.2.1.0) was used to perform statistical analysis. The relationship between the different conditions of RTX quantification was performed by a nonparametric regression. Passing–Bablok regression analysis [28] was performed to investigate any linear relationship between the methods. The regression equation (slope and intercept) was expressed with a 95% confidence interval. Method agreement was evaluated using Bland–Altman analysis [29]. The scatter of the result from the patient samples between the two methods was also shown. The numerical results were reported as mean ± 1.96 SD. The samples below the LLOQ were excluded, and *p* < 0.05 was considered statistically significant.

## 4. Conclusions

We described two completely validated MS methods of quantification of RTX in plasma. To our best knowledge, these methods are the first using full-length stable-isotope-labeled RTX as an internal standard. This approach may be considered as the most robust since the same labeled mAb undergoes all the analytical steps and mimic the mAb quantified. Both MS methods were robust and reliable in terms of analytical performances and could be applied to routine clinical samples.

## Figures and Tables

**Table 1:** Surrogate peptides pre-selected for Rituximab (RTX) quantification by LC-MS/HRMS using the full-length stable isotope-labeled rituximab (SIL-RTX). Peptides in bold were used for the quantification.

Compound	Peptide	Precursor Ion		Product Ion		
		(*m/z*)	Charge	Ion	(*m/z*)	Charge
**RTX**	**FSGS**	803.8890	+2	y8	926.4942	+1
				y10	1084.5629	+1
	**pQVQ**	980.5467	+2	y13	1252.7260	+1
				y6	558.3246	+1
	pQIVL	904.4936	+2	y11	1069.5888	+1
				y7	675.3308	+1
**SIL-RTX**	**FSGS**	808.8931	+2	y8 y10	936.5024 1094.5716	+1 +1
	**pQVQ**	988.5609	+2	y13 y6	1268.7544 566.3360	+1 +1
	pQIVL	908.5007	+2	y11 y7	1077.6030 683.3450	+1 +1

**Table 2:** Surrogate peptides pre-selected for Rituximab (RTX) quantification by LC-MS/MS using the full-length stable isotope-labeled rituximab (SIL-RTX). Peptide in bold was used for the quantification.

Compound	Peptide	Q1 ^1^ *m/z* (charge)	Q3 ^2^		CE ^3^ (eV)	CVt ^4^ (V)
			Ion	*m/z* (charge)		
**RTX**	**pQVQ**	**980.5 (+2)**	y6	558.3 (+1)	35	80
			y10	1027.6 (+1)		
			y12	1155.7 (+1)		
			y13	1252.7 (+1)		
	pQIVL	904.5 (+2)	y4	430.2 (+1)	20	90
			y7	675.3 (+1)		
			y9	788.4 (+1)		
			y11	1069.6 (+1)	30	
			y12	1156.6 (+1)		
**SIL-RTX**	**pQVQ**	**988.6 (+2)**	y6	566.3 (+1)	35	80
			y10	1043.6 (+1)		
			y12	1171.7 (+1)		
			y13	1268.7 (+1)		
	pQIVL	908.5 (+2)	y4	438.2 (+1)	20	90
			y7	683.3 (+1)		
			y9	796.4 (+1)		
			y11	1077.6 (+1)	30	
			y12	1164.6 (+1)		

^1^ Q1: first quadrupole; ^2^ Q3: third quadrupole; ^3^ CE: collision energy; ^4^ CVt: cone voltage.

**Table 3:** Inter-day validation of the determination of rituximab in plasma by LC-MS/HRMS using either FSGS or pQVQ proteotypic peptides. Data from seven calibration curves were prepared as a single replicate and analyzed on seven different days.

		FSGS			pQVQ	
Spiked (µg/mL)	Found (µg/mL) (mean ± s.d.)	Precision (%)	Accuracy (%)	Found (µg/mL) (mean ± s.d.)	Precision (%)	Accuracy (%)
10	9 ± 1	12.6	91.8	9 ± 2	16.6	88.4
25	26 ± 3	9.7	103.1	26 ± 3	11.8	102.8
50	51 ± 3	5.4	101.7	51 ± 5	9.1	102.0
100	98 ± 3	3.3	98.3	100 ± 6	6.0	100.0
150	150 ± 7	4.9	99.8	148 ± 8	5.6	98.6
200	200 ± 5	2.4	99.8	204 ± 5	2.6	102.1

s.d.: standard deviation.

**Table 4:** Inter-day validation of the determination of rituximab in plasma by LC-MS-MS using pQVQ as a surrogate peptide. Data from twelve calibration curves were prepared as a single replicate and analyzed on twelve different days.

	pQVQ		
Spiked (µg/mL)	Found (µg/mL) (mean ± s.d.)	Precision (%)	Accuracy (%)
5.0	5 ± 1	11.3	108.2
10.0	10 ± 1	6.2	103.1
20	20 ± 2	7.7	97.8
50	47 ± 4	9.2	94.1
100	96 ± 7	7.2	96.0
250	247 ± 16	6.7	98.6
500	510 ± 20	3.8	102.0

**Table 5:** Assessment of accuracy and precision for rituximab in plasma using the LC-MS/HRMS method and FSGS or pQVQ as surrogate peptides (*n* = 6, five days).

	Concentration (µg/mL)		Precision (%)		Accuracy (%)
Peptide	Spiked	Found (mean ± s.d.)	Within-Day	Between-Day	
FSGS	20	21 ± 2	8.4	7.3	106
	80	86 ± 7	5.3	6.9	107
	160	170 ± 13	7.3	0.5	106
pQVQ	20	22 ± 3	11.3	7.8	109
	80	84 ± 9	8.5	7.6	105
	160	168 ± 15	8.7	3.1	105

**Table 6:** Assessment of accuracy and precision for rituximab in plasma using the LC-MS/MS method and pQVQ as surrogate peptides (*n* = 3, ten days).

	Concentration (µg/mL)		Precision (%)		Accuracy (%)
Peptide	Spiked	Found (mean ± s.d.)	Within-Day	Between-Day	
pQVQ	15	16 ± 2	8.4	5.2	104
	75	76 ± 7	6.4	8.3	101
	300	291 ± 22	5.3	5.5	97

**Table 7:** Matrix effect calculated from six human plasmas spiked at 50 µg/mL (LC-MS/HRMS), or four human plasmas spiked at 15 and 300 µg/mL (LC-MS/MS).

Method	Peptide	Ion Suppression	Min; Max
LC-MS/HRMS	FSGS	−67%	−54%; −78%
	pQVQ	−11%	15%; −35%
	pQIVL	−93%	−88%; −95%
LC-MS/MS	pQVQ	−17%	0%; −34%
	pQIVL	−22%	1%; −44%

**Table 8:** Pre-analytical stability of rituximab at −20 °C over three months and autosampler stability (+4 °C, 72 h), calculated with the pQVQ peptide. Data obtained via LC-MS/MS assay.

Temperature	Concentration (µg/mL)		Precision (%)	Accuracy (%)
	Spiked	Found (mean ± s.d.)		
+4 °C	15	16 ± 1	7.9	107.5
	75	75 ± 2	3.2	101.0
	300	297 ± 6	2.0	98.8
−20 °C	15	14 ± 2	13.2	95.8
	75	72 ± 3	4.3	95.3
	300	319 ± 21	6.7	106.2

**Table 9:** Pre-analytical stability of rituximab at −20 °C over three months and autosampler stability (+4 °C, 72 h), calculated with the pQVQ peptide. Data obtained via LC-MS/MS assay.

Patient	FSGS			pQVQ		
	Found (µg/mL) (mean ± s.d.)	Repro. (%)	Diff. (%)	Found (µg/mL) (mean ± s.d.)	Repro. (%)	Diff. (%)
P1	100 ± 2	1.5	3.0	102 ± 7	6.5	9.5
P3	242 ± 25	10.3	−14.1	232 ± 15	6.4	−11.0
P5	244 ± 19	7.7	−12.2	229 ± 24	10.5	−6.8
P7	305 ± 8	2.7	4.6	286 ± 33	11.5	8.0
P9	<2	-	-	<2	-	-
P13	174 ± 13	7.7	−12.2	180 ± 7	4.1	−1.1
P15	139 ± 2	1.2	−1.8	147 ± 14	9.3	10.8

Repro.: Reproducibility; Diff.: Difference in concentration between the first and the forth dosage after three freezing/thawing cycles.

## Data Availability

The data presented in this study are available on request from the corresponding author.

## Sample Availability

Samples of the compounds are not available from the authors.

## Abbreviations

IgG	immunoglobulin G
CR3	complement receptor 3
Fcγ	constant fragment gamma
HC	heavy chain
LC	light chain
QVQ	peptide QVQLQQPGAELVKPGASVK
pQVQ	peptide QVQLQQPGAELVKPGASVK with pyroglutamination of N-terminal glutamine
ASGY	peptide ASGYTFTSYNMHWVK
pQIVL	peptide QIVLSQSPAILSASPGEK with pyroglutamination of N-terminal glutamine
FSGS	peptide FSGSGSGTSYSLTISR
GLEW	peptide GLEWIGAIYPGNGDTSYNQK
ASSS	peptide ASSSVSYIHWFQQKPGSSPKPWIYATSNLASGVPVR
VEAE	peptide VEAEDAATYYCQQWTSNPPTFGGGTK
STYY	peptide STYYGGDWYFNVWGAGTTVTVSAASTK
LLOQ	lower limit of quantification
PRM	parallel reaction monitoring
MRM	multiple-reaction monitoring
SD	standard deviation
QCs	quality controls
nSMOL	nano-surface and molecular-orientation limited
FA	formic acid
